# Effects of different doses of taurine supplementation on repeated-sprint performance after exhaustive exercise in a high temperature and humidity environment

**DOI:** 10.3389/fnut.2026.1766546

**Published:** 2026-05-20

**Authors:** Xiaodong Cheng, Yitong Lin, Zihao Li

**Affiliations:** 1 Blockchain and Healthcare, Health Service Research Center, Xi'an Medical University, Xi'an, China; 2 School of Kinesiology and Health, Capital University of Physical Education and Sports, Beijing, China; 3 Institute of Physical Education and Training, Capital University of Physical Education and Sports, Beijing, China

**Keywords:** college students, exhaustive exercise, high temperature and high humidity environment, repeated-sprint performance, taurine

## Abstract

**Objective:**

This study investigated the effects of acute pre-exercise taurine supplementation at different doses on exercise performance during exhaustive exercise and subsequent repeated sprint (RS; 6 sprints) activity in a high temperature and high humidity (HTHM) environment.

**Methods:**

Sixteen college students (8 males and 8 females) participated in a single-blind, randomized, crossover-controlled trial. Participants completed four experimental conditions: high-dose taurine (HDG, 6g), medium-dose taurine (MDG, 4g), low-dose taurine (LDG, 1g), and placebo (PG). Sports performance, physiological responses, and subjective perceptions were assessed, including peak power (PP), mean power (MP), fatigue index (FI), reverse vertical jump (RVJ) height, time to exhaustion (ET), heart rate (HR), blood lactate (BLA), and rating of perceived exertion (RPE).

**Results:**

During RS, PP and MP declined progressively across sprints (P < 0.05). Taurine supplementation attenuated the decline in power output compared with PG. In males, the HDG showed greater preservation of PP and MP, whereas in females, the LDG was more effective in maintaining MP, and the PG exhibited the greatest reduction in power output. Taurine supplementation increased exercise tolerance in a dose- and sex-dependent manner. ET was significantly longer in the HDG than in the PG in males, whereas in females, ET was significantly higher in the MDG compared with the PG (P < 0.05). RVJ performance decreased following exhaustive exercise in females (P < 0.05). Although no significant differences were observed in HR, BLA, or RPE among conditions, typical post-exercise response patterns were evident.

**Conclusion:**

Acute taurine supplementation prolongs time to exhaustion and enhances repeated sprint performance in HTHM environment, with dose- and sex-specific effects.

**Clinical Trial registration:**

ClinicalTrials.gov, NCT06824571, January 22, 2025. Retrospectively registered.

## 1 Introduction

A high temperature and high humidity (HTHM) environment is typically defined by an ambient temperature exceeding 32 C and relative humidity above 60% (1). As both temperature and humidity increase, declines in exercise performance and cognitive function are commonly observed (2). Exercise under such conditions elevates the body's heat load, imposing additional strain on thermoregulatory and fluid balance mechanisms (3). This physiological burden can disrupt pacing, reduce power output and volitional capacity, and accelerate the onset of fatigue (4, 5), ultimately leading to a marked impairment in overall performance. Accordingly, mitigating the adverse effects of heat and humidity and promoting effective recovery are critical for maintaining performance during prolonged or repeated exercise. In both training and competition, athletes are often required to continue exercising despite fatigue, making recovery following exhaustion particularly important for subsequent performance. Various preventive and recovery strategies have been proposed. External approaches, such as heat-adaptive clothing and specialized training garments, can facilitate adaptation to HTHM environments (6). In parallel, nutritional supplementation provides an effective internal strategy due to its practicality and capacity to support physiological regulation (7, 8). Among commonly used supplements, including caffeine, creatine, and taurine, taurine is of particular interest owing to its favorable safety profile, ease of use, and potential thermoregulatory effects—such as enhancing localized sweating responses (9). These characteristics make taurine a promising candidate for improving performance under HTHM conditions.

Taurine, accounting for approximately 50–60% of the free amino acid pool in mammalian tissues, is the most abundant sulfur-containing non-proteinogenic amino acid (10). It is involved in multiple physiological processes relevant to exercise performance. In skeletal muscle, taurine is present at high intracellular concentrations, and its uptake is mediated by the taurine transporter (TauT), which facilitates intracellular calcium handling within the sarcoplasmic reticulum, particularly in type II muscle fibers (11, 12). This modulation of calcium homeostasis has been associated with enhanced contractile function in both skeletal and cardiac muscle (13). Consistently, inhibition of taurine uptake in TauT knockout models accelerates fatigue onset (14), and taurine depletion has been shown to impair skeletal muscle function *in vitro* (15). Beyond its role in calcium regulation, taurine contributes to mitochondrial buffering capacity (15) and modulates neurotransmission through activation of γ-aminobutyric acid receptor subtypes in the thalamus (16), both of which may influence exercise performance. A growing body of evidence has examined the effects of taurine supplementation across different dosages. For example, Warnock et al reported that oral taurine supplementation at 50 mg·kg^−1^ body weight (~3.5–4.5 g) improved repeated sprint performance (17). Taurine has also been shown to attenuate oxidative stress and muscle damage during eccentric exercise (18) and may enhance anaerobic capacity (19). However, findings at lower doses are inconsistent. While 1 g of taurine improved 3-km running performance, doses of 1–1.6 g showed no significant effect during 4-km time trials or continuous endurance exercise (20). In contrast, short-term supplementation (6 g·day^−1^ for 7 days) has been reported to improve maximal oxygen uptake (VO_2_max) (21). Environmental stress appears to further modulate taurine's ergogenic effects. In HTMT conditions, taurine supplementation has been shown to enhance endurance performance (22). Acute taurine ingestion under heat stress increased time to exhaustion by approximately 10%, while reducing core temperature, perceived exertion in the later stages of exercise, and post-exercise blood lactate levels (23). Despite these findings, the dose–response relationship of acute taurine supplementation under combined heat and humidity stress remains insufficiently characterized, particularly in college populations. Exercise in HTHM environments imposes additional thermal and metabolic strain, which may alter taurine kinetics and modify the effective dose required for performance enhancement. Although taurine has demonstrated potential to improve repeated sprint (RS) ability, endurance performance, and post-exercise metabolic responses, its dose-dependent effects on repeated sprint performance following exhaustive exercise in hot and humid conditions remain unclear.

Accordingly, this study investigated the dose–response effects of acute taurine supplementation on RS performance following exhaustive exercise in the HTHM environment. We hypothesized that taurine would attenuate fatigue-related physiological disturbances and improve post-exhaustion RS performance.

## 2 Methods

### 2.1 Research design

This trial was registered at ClinicalTrials.gov (NCT06824571) on January 22, 2025 (retrospectively registered). Participant recruitment was conducted from September 2022 to October 1, 2022, and experimental testing took place between November 1 and December 20, 2022. Prospective registration was not feasible due to delays in submission; however, all primary and secondary outcomes were predefined prior to study initiation and remained unchanged throughout the study. The study was conducted in accordance with CONSORT guidelines. A randomized, single-blind, crossover-controlled design was employed. Sixteen participants were randomly assigned to four sequences (A–D), with each sequence comprising four participants. Each participant completed four experimental conditions, separated by a minimum 7-day washout period. The order of conditions was determined using computer-generated randomization.

Participants were blinded to supplement allocation. The placebo consisted of maltodextrin matched in mass to the taurine doses (1 g, 4 g, or 6 g) and dissolved in the same volume of water. To ensure effective blinding, both taurine and placebo beverages were prepared using identical sweeteners and served in opaque containers, rendering them indistinguishable in taste and appearance. All supplements were coded and administered in a standardized manner. Beverages were freshly prepared prior to ingestion and consumed under direct laboratory supervision to ensure full compliance. The experimental conditions included high-dose taurine (HDG, 6 g), medium-dose taurine (MDG, 4 g), low-dose taurine (LDG, 1 g), and placebo (PG) (24).

Taurine dosage was not normalized to body mass, and no weight-adjusted analysis was performed. Consequently, inter-individual variability in relative dosing may have influenced the results, particularly in sex-specific comparisons and dose–response interpretations. Although the crossover design incorporated randomized treatment sequences and a 7-day washout period, potential sequence, period, and carry-over effects were not formally assessed statistically. Therefore, residual confounding from these factors cannot be entirely excluded and should be considered when interpreting the findings.

**Figure 1:**
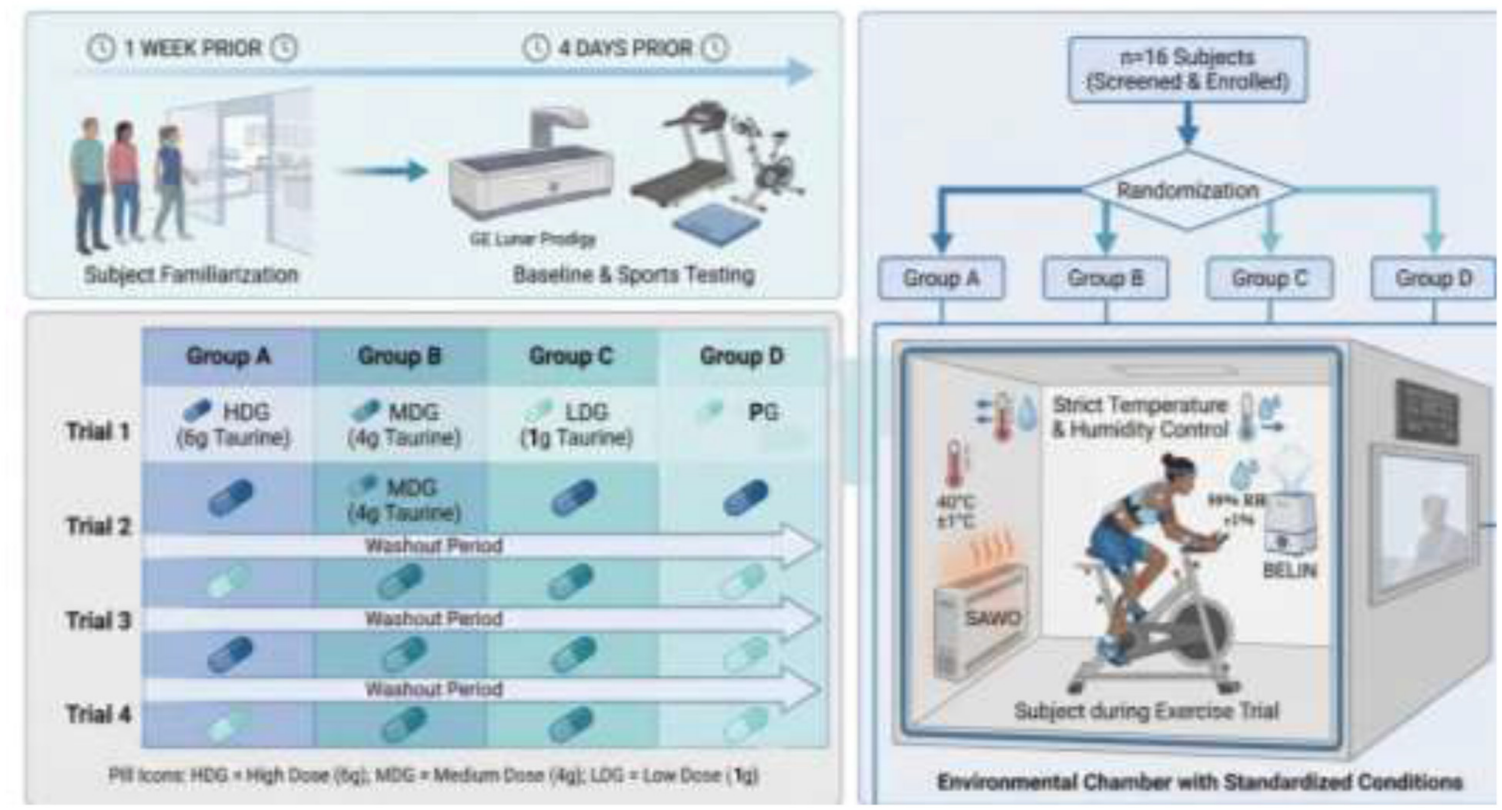
Randomized crossover study methodology to assess taurine dosage effects in an environmental chamber. Including participant recruitment, baseline measurements, randomization into taurine dose groups, and timeline of exercise testing and data collection.

Environmental conditions were tightly controlled using heating (SAWO, CON4, Finland) and humidification (BELIN, SC-G060ZS, China) systems, maintaining temperature and humidity within ±1 C of target values. Participants attended a familiarization session one week prior to the experiment to minimize learning effects. Four days before the first trial, body composition was assessed using dual-energy X-ray absorptiometry (DXA; Lunar Prodigy, GE, USA), and baseline exercise testing was performed. The randomized, single-blind, crossover-controlled trial design and overall experimental procedures of this study are shown in Figure 1.

### 2.2 Participants

Sample size was estimated using G^*^Power (Version 3.1.9.7; Franz Faul, University of Kiel, Germany) (25). Parameters were set at α = 0.05, statistical power (1–β) = 0.85, and effect size (F) = 0.25 for a repeated-measures design with four conditions and eight measurements. The analysis indicated a requirement of at least 28 observations within a repeated-measures framework, rather than 28 independent participants. Accordingly, 16 participants (8 males and 8 females) were recruited from Capital University of Physical Education and Sports, with each participant completing all four conditions in a randomized crossover design. The sample size was determined to support the overall analysis. Sex-stratified analyses were conducted on an exploratory basis and should be interpreted with caution due to the limited subgroup sample sizes and the use of ANOVA, which may constrain the reliability of interaction and subgroup inferences. Baseline characteristics are presented as mean ± standard deviation (Table 1). The study was conducted in accordance with the Declaration of Helsinki. All participants provided written informed consent after receiving a detailed explanation of the study procedures. The protocol was approved by the Institutional Ethics Committee of Capital University of Physical Education and Sports, Beijing, China (No. 2021A43). The selected taurine doses (1 g, 4 g, and 6 g) were chosen to represent low, moderate, and high levels of acute intake commonly reported in the literature, while ensuring feasibility within a crossover design. Given sex-related differences in body mass (Table 1), the estimated relative doses were approximately 12, 48, and 72 mg·kg^−1^ in males and 17, 67, and 100 mg·kg^−1^ in females. Although these values provide a general reference, dosing was not normalized to body mass, and no weight-adjusted analyses were performed. Therefore, variability in relative dosing may have influenced the observed dose–response relationships and cannot be excluded when interpreting the results.

**Table 1:** Basic information of experimental subjects.

	Age (y)	Height (cm)	Weight (kg)
Male	23.50 ± 2.78	180.50 ± 8.28	83.67 ± 15.64
Female	23.13 ± 2.61	166.76 ± 6.50	59.98 ± 3.14

Values are presented as mean ± standard deviation. Height in cm, weight in kg, age in years.

Given the potential discomfort associated with exercise in the HTHM environment, testing was terminated upon meeting predefined stopping criteria. The inclusion and termination criteria were as follows: (1) Inclusion criteria:1) Enrolled university students; 2) Aged 18–28 years; 3) Classified as low risk for exercise participation, as indicated by negative responses to all items on the Physical Activity Readiness Questionnaire (PAR-Q) (23); 4) No consumption of taurine-containing supplements within the preceding month. (2) Exclusion Criteria: 1) Failure to meet the inclusion criteria; 2) History of cardiovascular or respiratory disease, or any medical condition that may compromise exercise performance; 3) Positive response to any item on the Physical Activity Readiness Questionnaire (PAR-Q); 4) Consumption of taurine-containing supplements within the preceding month.

To minimize potential confounding from dietary intake and stimulant use, participants were instructed to maintain consistent dietary habits across all trials. Specifically, they were asked to replicate their dietary intake during the 24 h preceding each trial and to avoid foods that could influence performance, such as those high in caffeine or simple sugars. Participants were required to abstain from alcohol and strenuous physical activity for 24 h prior to each trial, and from caffeine, energy drinks, and other stimulants for at least 12 h before testing. Water intake was permitted *ad libitum*, and participants were instructed to arrive at the laboratory in a dehydrated state. To support compliance, participants received hydration guidelines and were reminded to maintain adequate fluid intake on the day preceding each trial.

### 2.3 Exercise procedures

#### 2.3.1 Warm-up preparation

Two hours prior to testing, the environmental chamber was activated, and temperature and humidity were continuously monitored to ensure stable conditions. All equipment was checked and prepared in advance. Upon arrival at the laboratory, participants consumed the assigned taurine or placebo beverage under investigator supervision. All beverages were ingested approximately 50 min before the exercise test. Participants then remained seated until subsequent measurements and the standardized warm-up were initiated. Anthropometric measurements (height and body mass) were recorded, and participant information was entered into the testing system. Baseline heart rate (HR), rating of perceived exertion (RPE), and blood lactate (BLA) were assessed prior to exercise.

#### 2.3.2 Warm-up

Participants completed a 5-min steady-state warm-up on a cycle ergometer at a constant workload of 100 W under hot and humid conditions. Immediately following the warm-up, reverse vertical jump (RVJ) performance was assessed.

#### 2.3.3 First exercise-exercise to exhaustion test

Participants performed an incremental exercise test on a cycle ergometer under hot and humid conditions. The protocol began at an initial workload of 50 W, with power output increased by 50 W every 3 min while maintaining a constant cadence of 60 revolutions per minute (rpm) until volitional exhaustion. Time to exhaustion (TTE) was recorded as the primary outcome. Exhaustion was defined when one or more of the following criteria were met: (1) inability to maintain the required power output despite verbal encouragement; (2) attainment of maximal heart rate (HRmax); or (3) a decline in cadence of ≥10 rpm sustained for more than 20s (26).

#### 2.3.4 Rest

Immediately after exhaustion, the rating of RPE and HR was recorded, and BLA was measured. These variables were subsequently reassessed at 3, 5, 10, and 15 min post-exercise to evaluate recovery kinetics. At 20 min post-exercise, HR and BLA were measured again, and RPE as well as RVJ performance were additionally assessed.

#### 2.3.5 Second exercise-repetitive sprint exercise test

The cycle ergometer resistance was set to 7.5% of each participant's body mass. Participants then completed six maximal-effort sprints on the cycle ergometer. Resistance was automatically applied once cadence reached 120 revolutions per minute (rpm). Each sprint was preceded by a 10-s countdown and followed by a 10-s passive recovery interval. During each sprint, peak power (PP), mean power (MP), and fatigue index (FI) were recorded.

**Figure 2:**
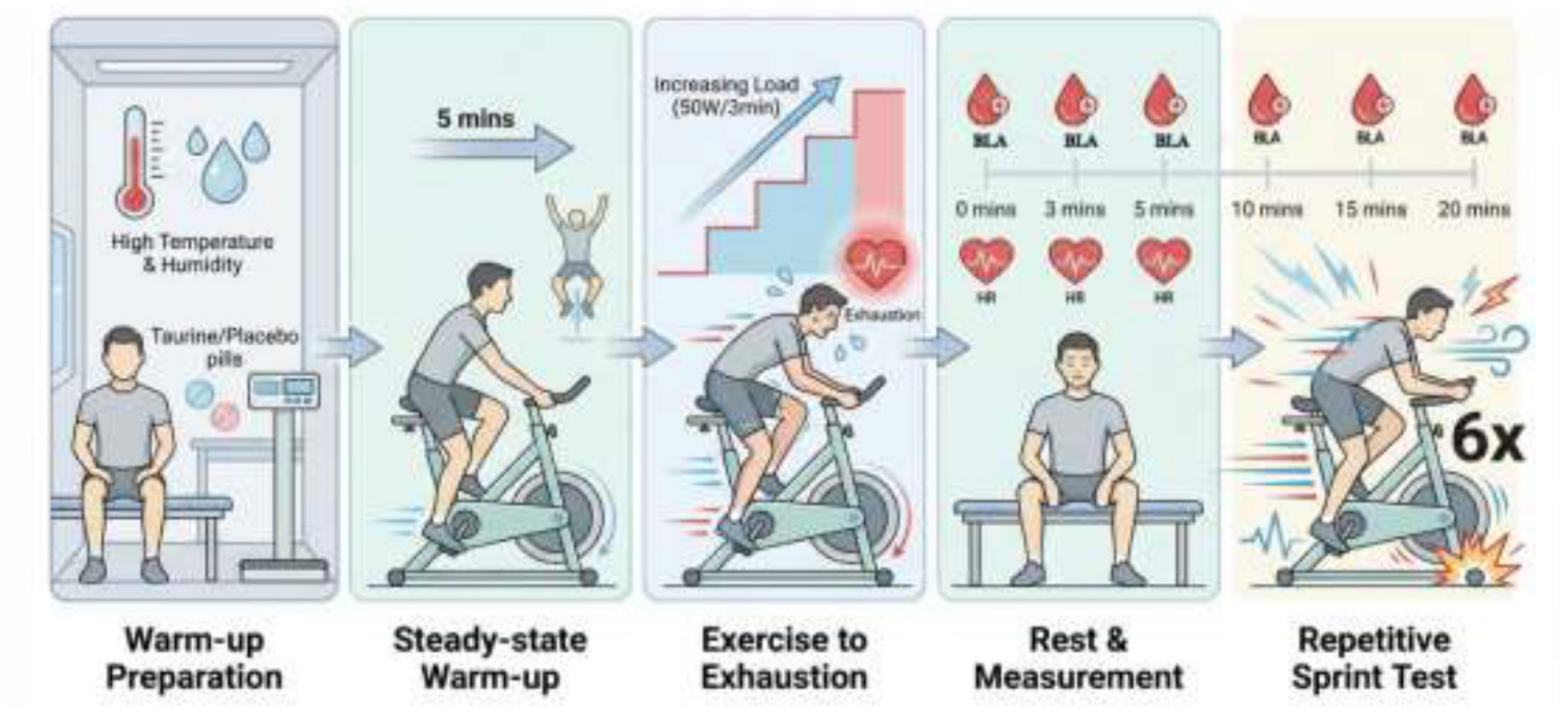
Detailed experimental procedure.

For the specific motion program, see Figure 2 below.

### 2.4 Indicators measurement

#### 2.4.1 Basic information

Participant characteristics, including name, age, sex, height, and body mass, were recorded. Height and body mass were measured using a calibrated audiometer and scale. Participants stood barefoot in an upright position at the center of the platform, with eyes facing forward, and were instructed to remain still and silent until measurements were displayed on the digital screen and recorded.

#### 2.4.2 Sports performance

##### 2.4.2.1 RS Exercise Performance

Subjects performed six 10-s Wingate anaerobic power tests using a Swedish Monark 839E power bicycle, with resistance set at 0.075 kg per kilogram of body weight (kg/kg-BW). The PP, MP, and FI were recorded following each RS.

##### 2.4.2.2 Reverse vertical jump (RVJ) height

The vertical jump test was performed using a Tapeswitch ControlMat™ jump mat (27). Participants stood with feet shoulder-width apart and initiated the test upon the investigator's “start” command. They performed a rapid countermovement squat until the knee joint reached approximately 90°, followed immediately by a maximal vertical jump. Each participant completed five trials, and the mean jump height was used for analysis.

##### 2.4.2.3 Exhaustion Time (ET)

The ET testing, a commonly used laboratory-based measure of exercise performance (28), was conducted using a Lode Corival cycle ergometer (Model 906900; Lode, Groningen, Netherlands). Participants began cycling at an initial workload of 50 W and continued following the predefined incremental protocol until volitional exhaustion. Time displayed on the ergometer at the point of exhaustion was recorded as ET. Immediately following the ET test, participants performed five maximal-effort vertical jumps using a standardized technique. Jump height was recorded for each trial, and the mean value was used for analysis.

#### 2.4.3 Physiological indicators

##### 2.4.3.1 Heart rate (HR)

The HR was continuously monitored in real time using a Polar H10 telemetry heart rate monitor (Polar Electro Oy, Finland). The chest strap was positioned at the level of the xiphoid process to ensure stable skin contact and minimize movement-related signal artifacts throughout exercise testing.

##### 2.4.3.2 Blood lactate (BLA)

The BLA was measured using a portable Lactate Scout 4 analyzer (EKF Diagnostics, Germany). The left earlobe was disinfected with an alcohol swab prior to capillary blood sampling. A small blood sample was then collected for BLA analysis (29). After measurement, the puncture site was gently compressed with sterile dry cotton to ensure hemostasis.

### 2.5 Subjective indicator

Subjective fatigue was assessed using the rating of the RPE scale. The RPE scale was displayed in the laboratory, and participants reported their perceived exertion based on their subjective sensations of fatigue at each corresponding time point during the experiment. The selected value was recorded by the investigator.

### 2.6 Statistical analysis

Data from this experiment were statistically analyzed using SPSS 27.0 software, with results expressed as mean ± standard deviation. Normality was assessed using the Shapiro–Wilk test together with residual diagnostics from the repeated-measures models. Paired-samples *t*-tests were used to compare pre-and post-test RVJ values. Due to the small sample size, the main analysis of this study employed repeated measures analysis of variance (ANOVA). For HR, BLA, RPE, PP, MP, and FI, repeated measures ANOVA (group × time) was conducted. Post hoc pairwise comparisons (within- group comparisons across sprints and between- group comparisons at each sprint) used Bonferroni correction. However, this method, based on ANOVA, cannot fully model the hierarchical and correlated structure of repeated measures in cross-designs as a linear mixed-effects model can. Therefore, although ANOVA is retained as a practical analytical method, this choice should be regarded as a methodological limitation, especially when interpreting interaction effects and exploratory gender stratified analyses. Additionally, a one-way ANOVA was performed for ET. These parametric procedures were applied under standard analytical assumptions and are commonly considered reasonably robust to modest departures from normality in balanced repeated-measures settings. Residual diagnostics did not indicate severe departures from normality; therefore, parametric analyses were retained. Effect sizes were expressed as η^2^partial, with values of 0.01, 0.06, and 0.14 representing small, medium, and large effect sizes, respectively. A significance level of *P* < 0.05 was used to determine statistically significant differences in this study. As part of the main analysis plan, there was no formal statistical assessment of sequence effects, periodic effect, and carry-over effects. Therefore, a comprehensive cross-effect analysis was not conducted, and when interpreting the research results, it was considered that this was a methodological limitation. The confidence intervals for the selected key pairwise comparisons were reported; however, in this analysis, confidence intervals were not consistently provided for all results and comparisons.

## 3 Results

### 3.1 Sports performance

#### 3.1.1 Peak power (PP)

**Figure 3:**
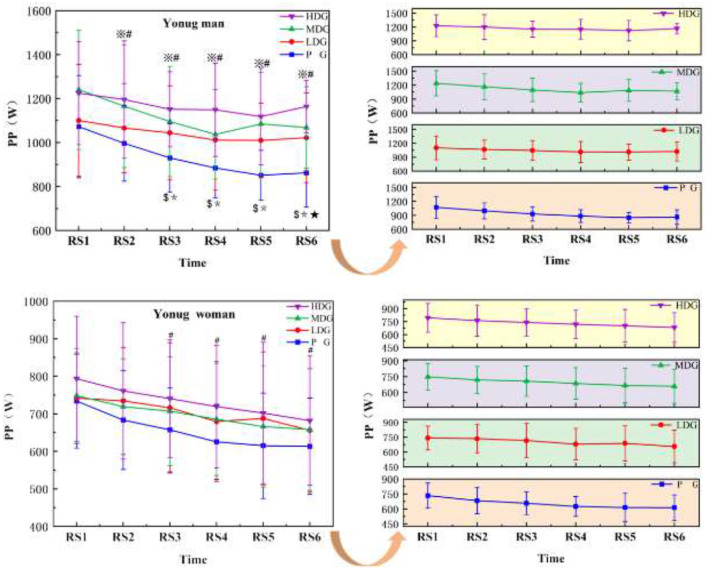
PP across RS for each taurine dose group in man and woman participants. Error bars represent ± 95% confidence intervals. Significance markers: ^*^P < 0.05, BL vs. HDG; ^$^*P* < 0.05, BL vs. MDG; ^※^*P* < 0.05, BL vs. LDG; ^#^*P* < 0.05, BL vs. PG; *P < 0.05,PG vs. HDG. Statistical analysis: repeated measures ANOVA with Bonferroni *post-hoc* test.

The results (Figure 3) showed a highly significant main effect of PP across each sprinting time point in males, *F*_(5, 24)_ = 44.96, *P* < 0.001, η^2^partial = 0.90. A significant interaction effect was observed between sprinting time point and dose, *F*_(5, 26)_ = 2.90, *P* = 0.03 < 0.05, η^2^partial=0.358. However, there was no significant main effect of dose on PP between dose groups, *F*_(3, 28)_ = 2.21, *P* = 0.11, η^2^partial = 0.19. As the number of sprints increased, all four groups of males showed a significant decrease in PP compared to BL. At the RS2, the LDG and PG began to show significant reductions in PP compared to BL. By RS3, the HDG and MDG also exhibited significant decreases in PP compared to BL. By RS6, all four groups demonstrated significant decreases in PP compared to BL. In terms of between-group comparison, PP was significantly higher in the HDG compared to the PG at RS6 [95%CI (137.88, 466.28), *P* < 0.05].

There was a significant main effect of PP at each time point of female (Figure 3) sprinting, *F*_(5, 24)_ = 6.23, *P* < 0.001, η^2^partial = 0.57. There was no significant interaction effect of sprinting time point^*^dose, *F*_(5, 26)_ = 1.19, *P* = 0.33, η^2^partial = 0.19, and there was no significant main effect of PP between dose groups, *F*_(3, 28)_ = 0.41, *P* = 0.74, η^2^partial = 0.04. By the RS6, there was a significant decrease in PP in the PG compared to the LDG at the RS1 [95%CI (−114.18, 176.29), *P* < 0.05].

#### 3.1.2 Mean power (MP)

**Figure 4:**
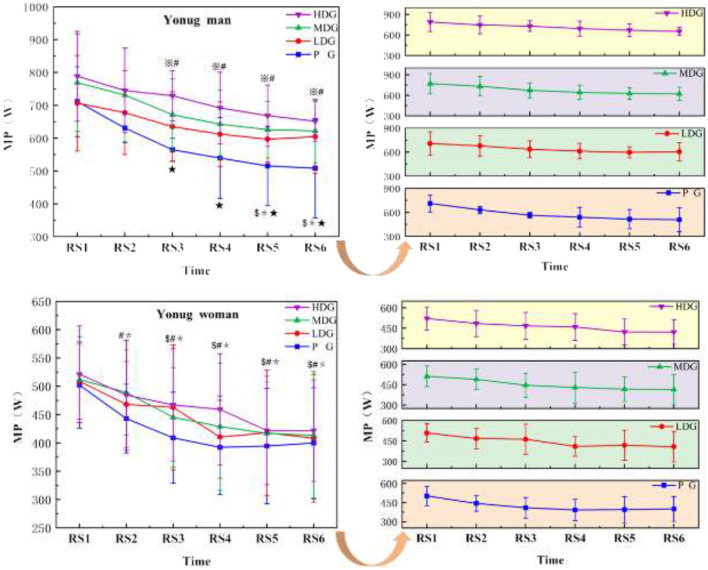
MP across RS for each taurine dose group in man and woman participants. Error bars represent ± 95% confidence intervals. Significance markers: ^*^P < 0.05, BL vs. HDG; ^$^*P* < 0.05,BL vs. MDG; ^※^*P* < 0.05, BL vs. LDG; ^#^*P* < 0.05, BL vs. PG; *P < 0.05,PG vs. HDG. Statistical analysis: repeated measures ANOVA with Bonferroni *post-hoc* test.

The results (Figure 4) indicated a highly significant main effect of MP across sprint time points in males, *F*_(5, 24)_ = 14.80, *P* < 0.001, η^2^partial=0.76. There was no significant interaction effect between sprint time point and dose, *F*_(5, 26)_ = 1.75, *P* = 0.15, η^2^partial = 0.25. However, a significant main effect of dose was observed, *F*_(3, 28)_ = 2.89, *P* = 0.047 < 0.05, η^2^partial = 0.23. As the number of sprints increased, all four groups of men showed a significant decrease in MP compared to BL. Significant decreases in MP relative to BL were observed in the LDG and PG starting at RS3. From RS5, both the MDG and HDG showed significant decreases in MP compared to BL. By RS6, all four groups exhibited significant decreases in MP relative to BL. In terms of between-group comparisons, the HDG showed a significantly higher MP than the PG at RS3 [95% CI (68.95, 291.78), *P* < 0.05], RS4 [95% CI (55.05, 362.89), *P* < 0.05], RS5 [95% CI (49.50, 430.06), *P* < 0.05], and RS6 [95% CI (97.43, 369.20), *P* < 0.05].

There was a highly significant main effect of MP across sprint time points for female (Figure 4), *F*_(5, 24)_ = 12.18, *P* < 0.001, η^2^partial = 0.72. No significant interaction effect was observed between sprint time point and dose, *F*_(5, 26)_ = 3.31, *P* = 0.02, η^2^partial=0.39, and there was no significant main effect of dose on MP between groups, *F*_(3, 28)_ = 0.32, *P* = 0.81, η^2^partial = 0.03. As the number of sprints increased, females in the HDG, MDG, and PG showed a significant decrease in MP compared to BL. Significant decreases in MP relative to BL were observed in the HDG and PG starting at RS2 [95%CI (−53.72, 137.05), *P* < 0.05], while the MDG showed a significant decrease beginning at RS3 [95%CI (−34.51,126.62), *P* < 0.05]. By RS6, the HDG, MDG, and PG experienced a significant decrease in MP compared to BL (*P* < 0.05).

#### 3.1.3 Fatigue index (FI)

**Figure 5:**
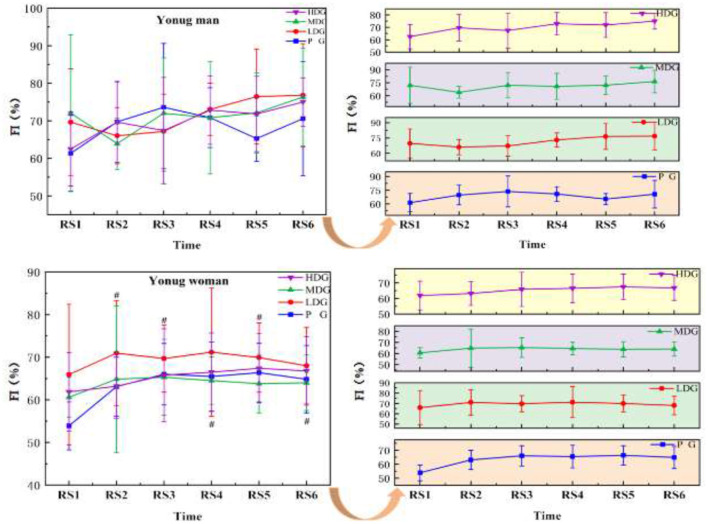
FI across six sprints for each taurine dose group in man and woman participants. Error bars represent ± 95% confidence intervals. Significance markers: ^#^*P* < 0.05, indicates comparison with BL in the PG. Statistical analysis: repeated measures ANOVA with Bonferroni *post-hoc* test.

The results (Figure 5) indicated a significant main effect of FI across sprint time points in males, *F*_(5, 24)_ = 3.04, *P* = 0.03 < 0.05, η^2^partial = 0.39. However, there was no significant interaction effect between sprint time point and dose, *F*_(5, 26)_ = 2.52, *P* = 0.06, η^2^partial=0.33, and no significant main effect of dose on FI between groups, *F*_(3, 28)_ = 0.33, *P* = 0.80, η^2^partial = 0.03. For females (Figure 5), however, there was no significant main effect of FI at the sprint time point, *F*_(5, 24)_ = 3.09, *P* = 0.02, η^2^partial = 0.10. Additionally, no significant interaction effect was found between sprint time point and dose, *F*_(5, 26)_ = 0.79, *P* = 0.65, η^2^partial = 0.08, and no significant main effect of dose on FI between groups, *F*_(3, 28)_ = 0.95, *P* = 0.43, η^2^partial = 0.09. As the number of sprints increased, the young woman PG showed a significant increase in FI compared to BL. At RS3 [95%CI (6.95, 11.45), *P* < 0.05] and RS6 [95%CI (8.48, 13.39), *P* < 0.05], the PG began to show a significant increase in FI compared to BL.

#### 3.1.4 Exhaustion Time (ET)

**Figure 6:**
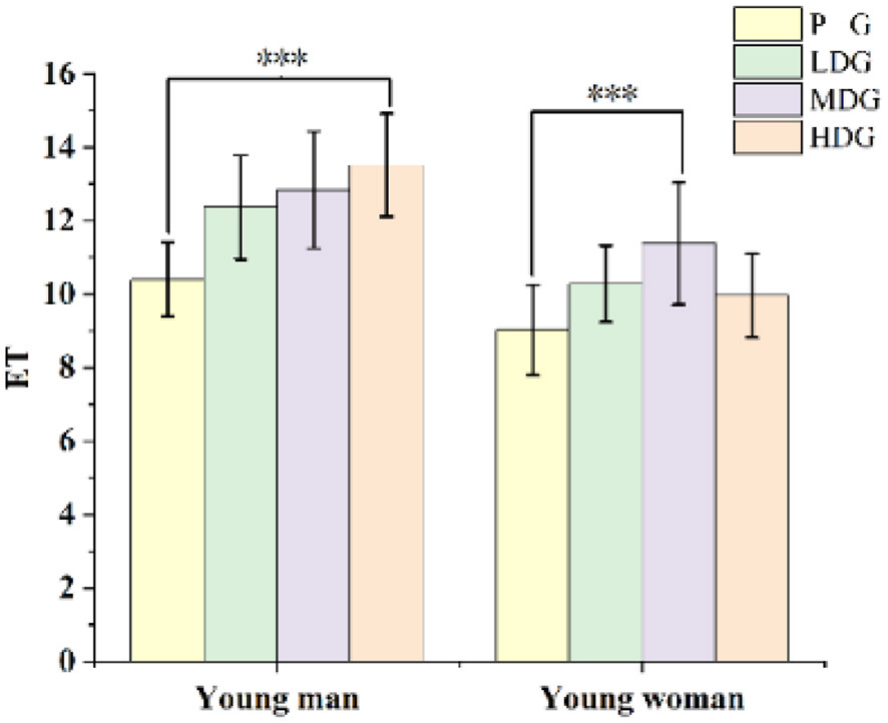
ET for each taurine dose group in man and woman participants. Error bars represent ± 95% confidence intervals. Significance markers: ^*^P < 0.05, indicates a significant difference compared to the group. Statistical analysis: one-way ANOVA with *post-hoc* multiple comparisons.

One-way ANOVA for ET (Figure 6), with different doses as a between-groups factor, revealed significant differences in ET among the different-dose groups for males, *F*_(3, 15.5)_ = 3.30, *P* < 0.05. *Post-hoc* multiple comparisons indicated that ET was significantly higher in the HDG than in the PG for male (*P* < 0.05). Similarly (Figure 6), for females, a one-way ANOVA showed significant differences in ET among dose groups, *F*_(3, 15.5)_ = 3.32, *P* < 0.05. *Post-hoc* comparisons revealed that ET was significantly higher in the MDG than in the PG for females [95%CI (−0.75, 2.71), *P* < 0.05].

#### 3.1.5 Reverse vertical jump value

##### 3.1.5.1 Statistical analysis: paired-samples *t*-test.

**Figure 7:**
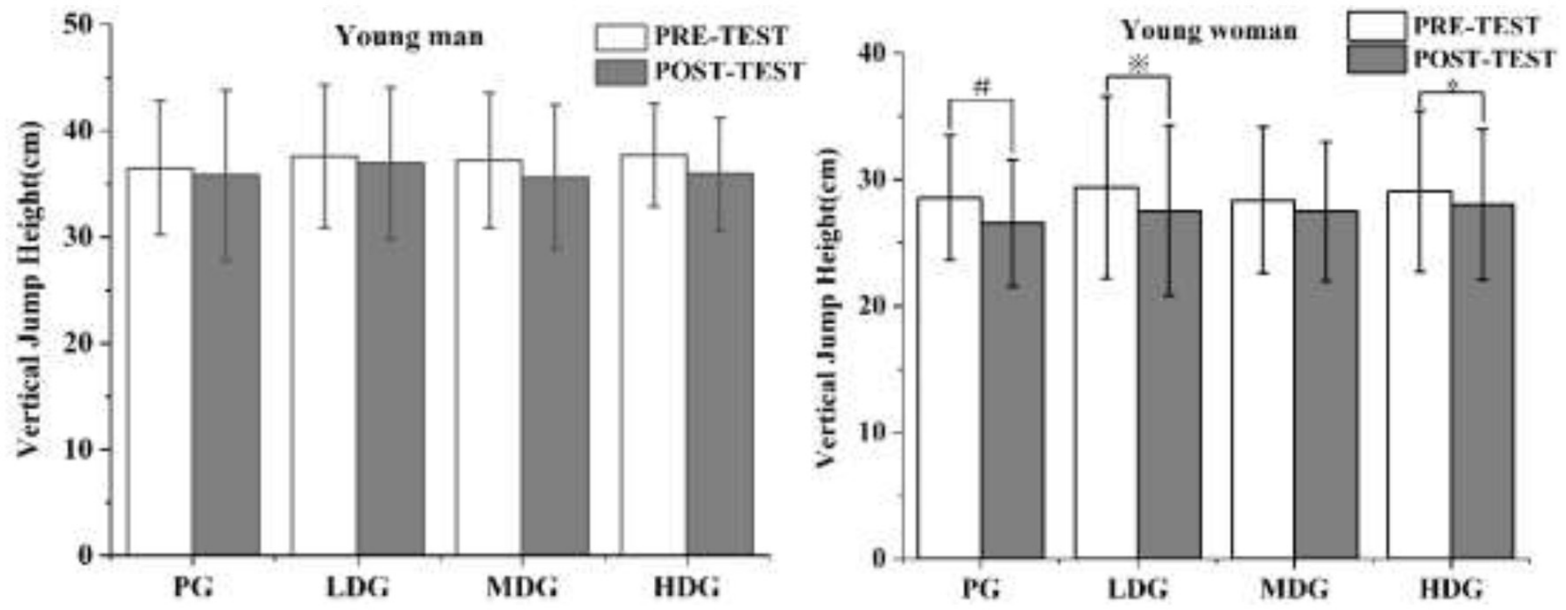
RVJ heights pre- and post-exercise for each taurine dose group in man and woman participants. Error bars represent ± 95% confidence intervals. Significance markers: *P < 0.05, indicates pre-exercise vs. post-exercise comparisons in the HDG. ※*P* < 0.05, indicates pre-exercise vs. post-exercise comparison in the LDG. #*P* < 0.05, indicates pre-exercise vs. post-exercise comparison in PG. Statistical analysis: paired-samples T-test.

A paired-samples *t*-test on **RVJ** values revealed no significant decrease before or after exercise in any of the four groups of males (Figure 7). In females (Figure 7), however, **RVJ** values were significantly lower pre-exercise than post-exercise in the HDG [95%CI (−8.26, 6.31), *P* < 0.05], LDG [95%CI (−10.10, 6.40), *P* < 0.05], and PG [95%CI (−7.39, 4.39), *P* < 0.05]. No significant differences were observed in the MDG female group. Overall, these results suggest that female participants experienced reductions in RVJ under certain dose groups, while male participants maintained consistent performance.

### 3.2 Physiological indicators

#### 3.2.1 Heart rate (HR)

**Figure 8:**
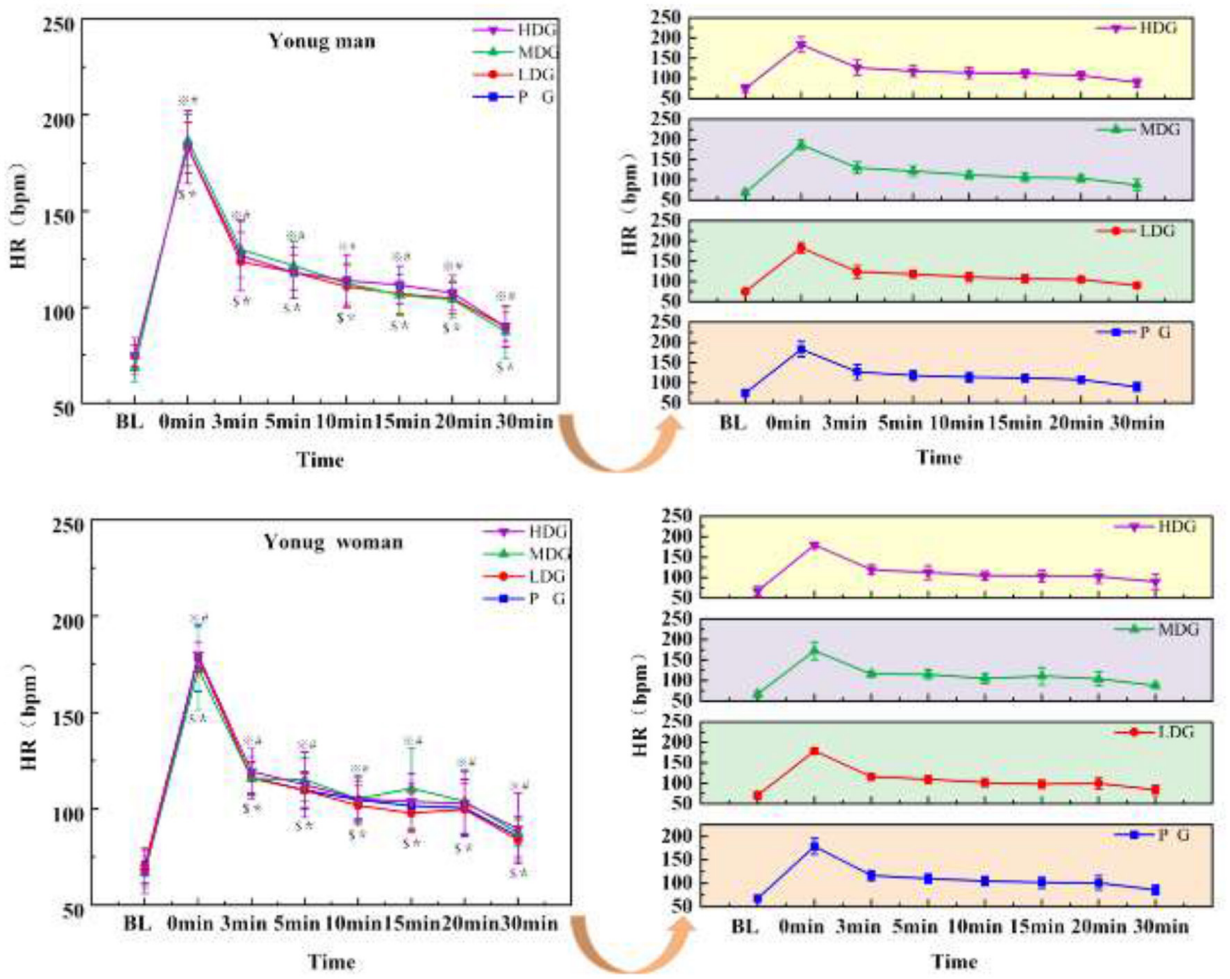
HR across eight time points for each taurine dose group in man and woman participants. Error bars represent ± 95% confidence intervals. Significance markers:^*^P < 0.05, indicates comparison with BL in the HDG. ^$^*P* < 0.05, indicates comparison with BL in the MDG. ^※^*P* < 0.05, indicates comparison with BL in the LDG. ^#^*P* < 0.05, indicates comparison with BL in the PG. Statistical analysis: repeated measures ANOVA with Bonferroni *post-hoc* test.

The results (Figure 8) showed a significant main effect of HR across eight time points for males, *F*_(7, 22)_ = 376.68, *P* < 0.001, η^2^partial = 0.99. There was no significant interaction effect between time point and dose, *F*_(7, 24)_ = 1.72, *P* = 0.15, η^2^partial = 0.34, and no significant main effect of dose on HR between groups, *F*_(3, 28)_ = 0.303, *P* = 0.823, η2partial = 0.031.

Similarly, for females (Figure 8), there was a significant main effect of HR across the eight time points, *F*_(7, 22)_ = 246.22, *P* < 0.001, η^2^partial = 0.99. No significant interaction effect was found between time point and dose, *F*_(7, 24)_ = 1.68, *P* = 0.16, η^2^partial = 0.32, and no significant main effect of dose on HR between groups, *F*_(3, 28)_ = 0.175, *P* = 0.913, η^2^partial = 0.018.

At 0 min and 30 min, a significant increase in HR compared to BL was observed in the HDG, MDG, LDG, and PG.

#### 3.2.2 Blood lactate (BLA)

**Figure 9:**
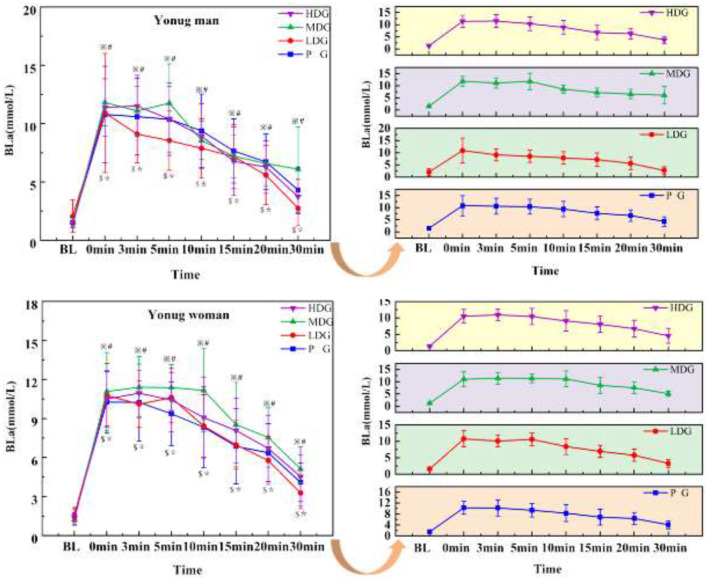
BLA concentrations across eight time points for each taurine dose group in man and woman participants. Error bars represent ±95% confidence intervals. Significance markers: ^*^P < 0.05 HDG vs. BL; ^$^*P* < 0.05 MDG vs. BL; ^※^*P* < 0.05 LDG vs. BL; ^#^*P* < 0.05 PG vs. BL. Statistical analysis: repeated measures ANOVA with Bonferroni *post-hoc* test.

The results (Figure 9) showed a significant main effect for BLA across the eight time points in males, *F*_(7, 22)_ = 41.39, *P* < 0.001, η^2^partial = 0.93. No significant interaction effect was found between time point and dose, *F*_(7, 24)_ = 2.19, *P* = 0.07, η^2^partial = 0.39, and there was no significant main effect of dose on BLA between groups, *F*_(3.28)_ = 0.63, *P* = 0.60, η^2^partial = 0.06. At 0 min and 20 min, HDG, MDG, LDG, and PG showed a highly significant increase in BLA compared to BL (*P* < 0.05).

There was a highly significant main effect for BLA across the eight time points in females (Figure 9), *F*_(7, 22)_ = 94.08, *P* < 0.001, η^2^partial = 0.97. No significant interaction effect was found between time point and dose, *F*_(7, 24)_ = 1.09, *P* = 0.40, η^2^partial = 0.24, and there was no significant main effect of dose on BLA between groups, *F*_(3, 28)_ = 0.919, *P* = 0.445, η^2^partial = 0.09. At 0 min and 30 min, HDG, MDG, LDG, and PG showed a highly significant increase in BLA compared to BL (*P* < 0.05).

## 4 Subjective indicators

**Figure 10:**
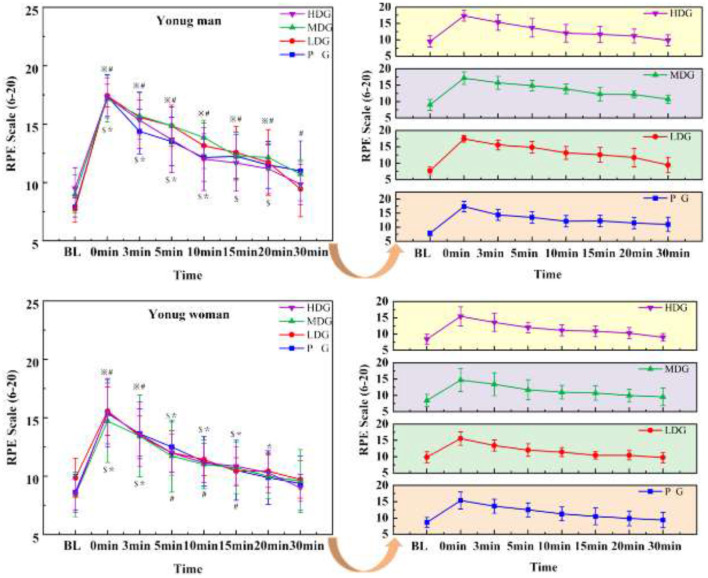
RPE across eight time points for each taurine dose group in man and woman participants. Error bars represent ± 95% confidence intervals. Significance markers:^*^P < 0.05, indicates comparison with BL in the HDG. ^$^*P* < 0.05, indicates comparison with BL in the MDG. ^※^*P* < 0.05, indicates comparison with BL in the LDG. ^#^*P* < 0.05, indicates comparison with BL in the PG. Statistical analysis: repeated measures ANOVA with Bonferroni *post-hoc* test.

The results (Figure 10) showed a highly significant main effect of RPE across the eight time points in males, *F*_(7, 22)_ = 74.16, P < 0.001, η^2^partial = 0.96. There was a significant interaction effect between time point and dose, *F*_(7, 24)_ = 2.44, *P* = 0.049 < 0.05, η^2^partial = 0.42, but no significant main effect of dose on RPE between groups, *F*_(3, 28)_ = 0.521, *P* = 0.671, η^2^partial = 0.053. At 0 min and 10 min, the HDG, MDG, LDG, and PG showed a highly significant increase in BLA compared to BL (*P* < 0.05). Starting at 15 min, the significant increase in BLA for the HDG compared to BL disappeared. By 30 min, only the PG continued to show a significant increase in RPE compared to BL (*P* < 0.05)

There was a highly significant main effect of RPE across the eight time points in females (Figure 10), *F*_(7, 22)_ = 23.04, *P* < 0.001, η^2^partial = 0.88. No significant interaction effect was observed between time point and dose, *F*_(7, 24)_ = 0.890, *P* = 0.529, η^2^partial = 0.206, and there was no significant main effect of dose on RPE between groups, *F*_(3, 28)_ = 0.266, *P* = 0.849, η^2^partial = 0.028. At 0 min, the HDG, MDG, LDG, and PG showed a highly significant increase in BLA compared to BL (*P* < 0.05). By 20 min, only the PG continued to show a significant increase in RPE compared to BL (*P* < 0.05).

## 5 Discussion

In this study, acute taurine supplementation demonstrated ergogenic effects in college students exercising under HTHM conditions. Taurine significantly prolonged the time to exhaustion and contributed to the maintenance of PP and MP across repeated sprints. RVJ performance following exercise exhibited sex-specific responses, while FI increased progressively across sprints, reflecting cumulative fatigue. In addition, taurine supplementation was associated with a reduction in subjective fatigue, as indicated by lower ratings of RPE during recovery. Although prior studies have suggested that taurine may influence cardiovascular and metabolic regulation, the present study did not assess key mechanistic variables. Therefore, any mechanistic interpretation should be made with caution and considered speculative.

### 5.1 Sports performance impact

In repeated sprint exercise, PP reflects the maximal power output achieved during the initial phase of each sprint and serves as an indicator of the muscle's capacity for rapid force production. In males, PP declined earlier in the LDG and PG than in the HDG and MDG groups, suggesting that higher taurine availability may contribute to the maintenance of power output across successive sprints. By RS6, PP in the HDG was significantly higher than in the LDG and PG. In females, the PG exhibited a significant decline in PP from RS3 to the end of the exercise relative to BL, whereas no significant reductions were observed in the taurine-supplemented groups. This pattern is consistent with previous findings (30), indicating a potential protective effect of taurine on high-intensity performance. Overall, PP decreased progressively with increasing sprint repetitions, which is consistent with the accumulation of fatigue and consequent reductions in power output (31). Heat stress HTHM environments further accelerate fatigue development and impair performance (32). Taurine may counteract these effects through several mechanisms, including modulation of thermoregulation, attenuation of exercise-induced oxidative stress, and protection against muscle damage (33, 34). In addition, taurine has been implicated in optimizing muscle energy metabolism, potentially through enhanced fatty acid oxidation, which may support sustained energy supply during repeated high-intensity efforts (20). While the present findings support an acute ergogenic effect of taurine, chronic supplementation may confer additional benefits for muscle function and endurance capacity (20). Taken together, these results suggest that taurine supplementation helps preserve muscle function and attenuate the decline in PP during repeated sprints following exhaustive exercise in hot and humid conditions (35).

The MP represents the average power output during maximal effort in the Wingate anaerobic test and is commonly used as an index of anaerobic capacity and fatigue resistance. In the present study, in males, the decline in MP occurred later in the HDG and MDG taurine groups compared with the LDG and PG. From RS3 to the end of the protocol, MP in the HDG remained significantly higher than in the PG, suggesting that higher taurine doses may be more effective in preserving MP during repeated sprint exercise, consistent with previous findings (36). In females, the temporal pattern differed. The MDG exhibited a delayed decline in MP compared with the HDG and PG, indicating that a moderate dose may be sufficient to attenuate fatigue-related reductions in MP. Notably, the LDG did not show a statistically significant decrease in MP across the protocol, suggesting that lower taurine doses may already be adequate to confer performance benefits in females. This observation raises the possibility of sex-specific dose–response characteristics, whereby higher doses do not provide additional advantages in women (37). Overall, MP decreased progressively with sprint repetition, reflecting cumulative fatigue under high-intensity and heat-stress conditions. Taurine may mitigate this decline through multiple mechanisms, including thermoregulatory effects and attenuation of metabolic disturbances associated with high-intensity exercise (21). In addition, taurine has been reported to influence calcium handling in the sarcoplasmic reticulum, potentially enhancing myofilament sensitivity and contractile function in both type I and type II muscle fibers (9), which may contribute to the preservation of MP during repeated sprints. While acute taurine supplementation appears to confer immediate benefits, evidence from chronic supplementation studies suggests that longer-term intake may further enhance muscle function and endurance capacity, potentially augmenting the effects observed in the present study.

The FI reflects the rate of performance decline during high-intensity exercise and is typically calculated from the decrement in performance between the initial and final exercise phases, thereby indicating the extent of fatigue development over repeated efforts. In males, FI fluctuated across sprint stages and showed an overall increasing tendency with repeated sprints; however, no statistically significant changes were observed, which is consistent with previous findings (38). In females, FI exhibited a gradual upward trend with increasing sprint repetitions, but no significant changes were detected in the taurine-supplemented groups. In contrast, the placebo group demonstrated a significant increase in FI relative to baseline, suggesting that taurine supplementation may attenuate the progression of fatigue in females. This sex-specific pattern is in line with previous reports suggesting that taurine may exert more pronounced anti-fatigue effects in females, potentially due to differences in hormonal profiles, muscle fiber composition, and metabolic regulation (39).Such differences may influence substrate utilization and fatigue-related metabolite accumulation during high-intensity exercise, thereby contributing to a reduced rate of performance decline (21). Additionally, taurine has been proposed to mitigate exercise-induced lactate accumulation and muscle fatigue (21), which may help stabilize performance during repeated sprint exercise in HTHM environments. High temperature and high humidity conditions are known to accelerate the development of peripheral and central fatigue and impair exercise performance (32). Taurine may counteract these effects through its roles in thermoregulation, membrane stabilization, and attenuation of exercise-induced muscle damage and fatigue (33). Overall, FI increased progressively with repeated sprinting, indicating cumulative fatigue and a gradual decline in repeated sprint performance following exhaustive exercise. The relatively higher initial FI values observed in taurine-supplemented conditions may be attributable to improved initial exercise performance capacity, leading to greater absolute performance output and thus a larger calculated fatigue-related decrement (21).

The ET refers to the duration an athlete can sustain exercise at a given intensity until volitional exhaustion. It is a comprehensive indicator of endurance performance, reflecting integrated cardiorespiratory function, muscular endurance, and energy metabolism. In the present study, males demonstrated a significant prolongation of ET following high-dose taurine supplementation, whereas females exhibited the greatest improvement in ET under medium-dose supplementation. These effective doses are slightly higher than those commonly reported in the literature, where taurine is typically administered at approximately 50 mg·kg^−1^ body mass. This discrepancy may be related to the specific physiological demands of exercise performed under high temperature and high humidity conditions. Heat stress increases metabolic demand and accelerates energy expenditure, thereby imposing greater strain on thermoregulatory and metabolic systems. This may alter taurine distribution and utilization, potentially necessitating higher effective doses to elicit ergogenic benefits in HTHM environments. The present findings are consistent with previous studies reporting that taurine supplementation at approximately 50 mg·kg^−1^ body mass can prolong time to exhaustion by~10% (9). In this study, taurine similarly extended ET under heat-stress conditions. Several mechanisms have been proposed to explain these effects, including regulation of intracellular calcium handling, attenuation of oxidative stress, and modulation of mitochondrial function, all of which may contribute to improved exercise tolerance under thermal stress. However, these variables were not directly assessed in the present study; thus, mechanistic interpretations should be considered speculative. In addition, taurine's thermoregulatory properties may further contribute to improved exercise performance under high temperature and high humidity conditions (40).

The RVJ test is a practical measure of neuromuscular fatigue and short-term recovery capacity (41). The RVJ test is a practical measure of neuromuscular fatigue and short-term recovery capacity (42), which may in turn support explosive performance such as vertical jumping. In the present study, males showed no significant change in RVJ height following exhaustive exercise, which is consistent with previous findings by Scott Forbes et al., reporting no significant effect of acute taurine supplementation on vertical jump performance (41). In contrast, females exhibited a significant reduction in RVJ height after the exhaustive exercise protocol. This observation is consistent with prior studies demonstrating exercise-induced decrements in vertical jump performance following high-intensity efforts (43). The decline in jump performance is generally attributed to neuromuscular fatigue, particularly impairments in excitation–contraction coupling and reduced muscle contractile efficiency. Following intense cycling exercise, disruption of calcium ion homeostasis within skeletal muscle may occur, leading to reduced calcium release from the sarcoplasmic reticulum during depolarization (44). This reduction in intracellular calcium availability can impair actin–myosin cross-bridge cycling, ultimately decreasing force production capacity in the lower limbs (45).

### 5.2 Changes in physiological indicators

After exhaustive exercise in the HTHM environment, HR exhibited significant time-dependent changes. HR increased rapidly immediately post-exercise, reaching its peak at exhaustion, and then gradually declined during recovery. However, HR did not return to pre-exercise levels even 30 min after exercise, which is consistent with previous reports (46). Other studies have similarly shown that HR may remain elevated for up to 60 min post-exercise, with no significant differences observed between experimental conditions (47). The delayed recovery of HR is closely related to exercise intensity and duration, as both factors substantially influence autonomic and cardiovascular recovery kinetics (48). In addition, exhaustive exercise under heat stress conditions induces marked alterations in autonomic nervous system balance (49), characterized by enhanced sympathetic activation and reduced parasympathetic reactivation (50). This autonomic imbalance contributes to sustained post-exercise tachycardia and delayed HR recovery.

BLA is closely associated with exercise intensity and peripheral muscle fatigue. During repeated sprint exercise, energy demand is primarily met through anaerobic glycolysis, resulting in lactate accumulation. In the present study, BLA increased immediately after exhaustive exercise and then gradually declined during recovery. In males, BLA returned to baseline levels by 30 min post-exercise, whereas in females, BLA remained elevated and did not fully return to baseline within the same recovery period. No significant differences were observed among the four experimental groups, indicating that acute taurine supplementation did not significantly alter post-exercise BLA dynamics under high temperature and high humidity conditions. These findings are generally consistent with previous studies reporting sustained post-exercise elevation of BLA during early recovery. For example, Gmada et al. (51) observed that BLA in males had not returned to pre-exercise levels at 20 min post-exercise, and other studies have reported persistently elevated lactate concentrations up to 15 min following high-intensity exercise (52). However, contrasting evidence suggests that acute taurine supplementation may facilitate faster lactate clearance under heat-stress conditions in some male cohorts, indicating that its effects on lactate metabolism may be context-dependent. In females, BLA remained elevated above baseline at 30 min post-exercise, which is consistent with reports showing that lactate levels may require up to approximately 50 min to return to resting values following high-intensity exercise (53). This delayed recovery may reflect sex-related differences in metabolic regulation, lactate transport, and clearance capacity. Specifically, males typically exhibit a higher proportion of type II muscle fibers, which are associated with greater lactate production during high-intensity efforts. In contrast, females generally have a relatively higher proportion of type I fibers, which are more oxidative but may exhibit different lactate transport and clearance dynamics during recovery (54). These physiological differences may contribute to the observed sex-specific patterns in post-exercise lactate recovery.

### 5.3 Subjective perception changes

The RPE is a widely used integrative indicator of physiological and psychological stress, commonly assessed using the Borg 6–20 scale, ranging from “very, very light” to “maximal exertion,” and reflecting an individual's subjective perception of effort and fatigue. In the present study, RPE increased rapidly immediately after exhaustive exercise and then gradually declined during recovery, returning to baseline levels by 30 min post-exercise. In males, the HDG exhibited the earliest recovery of perceived fatigue, with RPE returning to pre-exercise levels by approximately 15 min. In contrast, the PG demonstrated the slowest recovery, with RPE remaining elevated at 30 min post-exercise. This pattern suggests that taurine supplementation may facilitate a faster reduction in perceived exertion during recovery under heat-stress conditions. In females, RPE in the HDG showed a slightly delayed decline but still returned to baseline by 30 min post-exercise. The observed sex-related differences in recovery kinetics may reflect underlying physiological differences in fatigue perception and recovery regulation, potentially influenced by sex hormones such as estrogen, which has been reported to exert antioxidant and membrane-stabilizing effects that may attenuate exercise-induced oxidative stress and fatigue perception. Exhaustive exercise in HTHM environments induces substantial metabolic stress, including accumulation of metabolic byproducts and depletion of intramuscular energy substrates such as phosphocreatine and glycogen, which together contribute to elevated perceived exertion. In addition, HTHM environmental heat stress imposes both physiological and psychological strain, further amplifying subjective fatigue (3). During recovery, gradual clearance of metabolites and restoration of energy homeostasis contribute to the progressive reduction in RPE (55). Although taurine has been proposed to modulate oxidative stress, membrane stability, and energy metabolism, its precise contribution to subjective fatigue recovery in the present study remains indirect and should be interpreted cautiously, as mechanistic variables were not directly measured.

## 6 Limitations

Although the crossover design helps reduce inter-individual variability, the overall sample size in this study remains relatively limited. After stratification by sex, each subgroup consisted of only eight participants (*n* = 8), which may reduce statistical power and limit the robustness of subgroup analyses. In addition, confidence intervals were reported only for selected comparisons rather than systematically across all outcomes. This represents a limitation in reporting consistency, as it may reduce the comparability and transparency of effect size precision across different variables. Dietary intake and hydration status prior to each trial were controlled through participant instructions but were not objectively monitored or standardized. Given that exercise performance under high temperature and high humidity conditions is highly sensitive to hydration and nutritional status, uncontrolled variability across testing sessions may have influenced exercise tolerance and repeated sprint performance. Furthermore, the primary analyses did not include formal statistical evaluation of sequence, period, or carry-over effects. Although randomization and a 7-day washout period were implemented, the absence of explicit testing for these crossover-specific effects remains a methodological limitation and may affect the internal validity of the findings. Finally, the study was conducted under a single controlled high temperature and high humidity condition, which limits the generalizability of the results to other environmental settings. Future studies should incorporate formal assessment of crossover effects, increase sample size, improve objective monitoring of dietary and hydration status, and further explore taurine dose–response relationships across a broader range of environmental conditions.

## 7 Conclusion

In a HTHM environment, acute taurine supplementation was associated with longer ET and better maintenance of power output during subsequent RS exercise in college students. No clear between-condition differences were observed for HR, BLA, or RPE.

## Data Availability

The raw data supporting the conclusions of this article will be made available by the authors, without undue reservation.
